# Using HHsearch to tackle proteins of unknown function: A pilot study with PH domains

**DOI:** 10.1111/tra.12432

**Published:** 2016-10-09

**Authors:** David R. Fidler, Sarah E. Murphy, Katherine Courtis, Pantelis Antonoudiou, Rana El‐Tohamy, Jonathan Ient, Timothy P. Levine

**Affiliations:** ^1^ Department of Cell BiologyUCL Institute of OphthalmologyLondonUK

**Keywords:** GRAM domains, Gyp7p, pleckstrin homology (PH) domains, profile‐profile search, Saccharomyces cerevisiae, secondary structure prediction, structural bioinformatics, TBC1D15, Vid27p, Vps13p, YJL016W, YJL181C, YJR030C

## Abstract

Advances in membrane cell biology are hampered by the relatively high proportion of proteins with no known function. Such proteins are largely or entirely devoid of structurally significant domain annotations. Structural bioinformaticians have developed profile‐profile tools such as HHsearch (online version called HHpred), which can detect remote homologies that are missed by tools used to annotate databases. Here we have applied HHsearch to study a single structural fold in a single model organism as proof of principle. In the entire clan of protein domains sharing the pleckstrin homology domain fold in yeast, systematic application of HHsearch accurately identified known PH‐like domains. It also predicted 16 new domains in 13 yeast proteins many of which are implicated in intracellular traffic. One of these was Vps13p, where we confirmed the functional importance of the predicted PH‐like domain. Even though such predictions require considerable work to be corroborated, they are useful first steps. HHsearch should be applied more widely, particularly across entire proteomes of model organisms, to significantly improve database annotations.

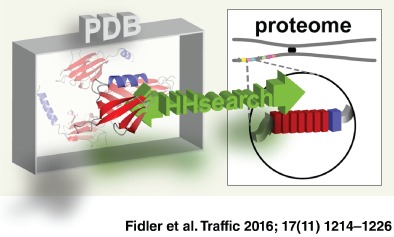

## 1 INTRODUCTION

An important way to determine the function of an unknown protein is to identify homology to a protein with known function. However, a significant minority (~20%) of protein sequences shows no obvious homology at the level of primary structure (sequence alone) to any other protein.^1^ Tools such as Basic Local Alignment Search Tool (BLAST) begin to perform poorly when sequence identities within a single domain drop below 30%.^2^ To overcome this problem, sequence‐sequence searches have been supplemented with profile‐sequence searches such as *PSI‐BLAST*,^3^ which increase sensitivity by searching with a profile that contains statistical information from a multiple sequence alignment (*MSA*) created from the initial *query*. Even more sensitive tools carry out profile‐profile searches, which extract family‐wide information for both query and *target*.^4^, ^5^, ^6^ Such tools involve the construction of profiles for all members of target sequence libraries, which requires considerable computational effort. Therefore, profile‐profile searches tend to be restricted to a small range of targets, most often the Protein Databank of solved structures (PDB). Profiles can implicitly encode aspects of secondary structure,^7^ but some tools go further by explicitly including secondary structure,^8^, ^9^, ^10^ either relying on solved structures or predicting secondary structural elements.^11^, ^12^ There has been a steep decline in the discovery of completely new folds,^6^ which suggests that many regions of unknown function will turn out to be distant structural homologues of known domains. It may be possible to detect these distant structural homologies by applying the most sensitive tools to all protein regions of unknown function. *HHsearch* is a powerful profile‐profile alignment tool^13^ that has not been applied widely or systematically.

Pleckstrin homology (PH) domains and their structural homologues, PH‐like domains, are one of the most common folds in eukaryotes.^14^ Although PH‐like domains have no unified function, they have typical docking sites for other proteins or inositide lipids.^15^ They are often involved in targeting peripheral membrane proteins involved in traffic and signaling.^16^ Hence, prediction of new PH‐like domains may identify residues that are functionally important for intracellular traffic. Since the initial discovery of classical PH domains, newly solved PH‐like structures have unexpectedly been found, both early on,^17^ and at least 10 times since.^18^, ^19^, ^20^, ^21^, ^22^, ^23^, ^24^, ^25^, ^26^, ^27^ Each of these discoveries added a new family of PH‐like domains to a growing PH‐like *clan*.^28^ PH‐like domains are therefore a good example of a structural fold with high sequence divergence.

The model organism *S. cerevisiae* is one of the most highly studied, especially in systems biology. Yet for 10%‐13% of yeast proteins there is no functionally or structurally relevant domain information (TL, unpublished observation). Progress in finding functions for yeast proteins with no recognizable domain is slower than expected.^1^ As an example that can be worked through in depth, we used HHsearch to study the PH‐like domain clan (sometimes called superfamily) in yeast. After bench‐marking HHsearch against PH‐like domains with solved structures, we searched for PH‐like domains in yeast. HHsearch was more accurate than tools used to annotate databases, and it detected some new domains easily. We found even greater sensitivity by seeding searches with yeast protein sequences, although this required high volume automated offline searches. Overall, we discovered 16 new yeast PH‐like domains. One of these is at the C‐terminus of Vps13p, a conserved membrane contact site protein implicated in sorting along the endocytic pathway. The presence of this one PH‐like domain was verified by demonstrating both that it has a specific targeting activity and that it is required for one aspect of the function of full length Vps13p. This study of PH‐like domains in yeast shows how the problem of proteins without domain information could be significantly reduced by systematic application of HHsearch.

## 2 RESULTS AND DISCUSSION

### 2.1 Curating PH‐like domains: what we knew already

**Figure 1:**
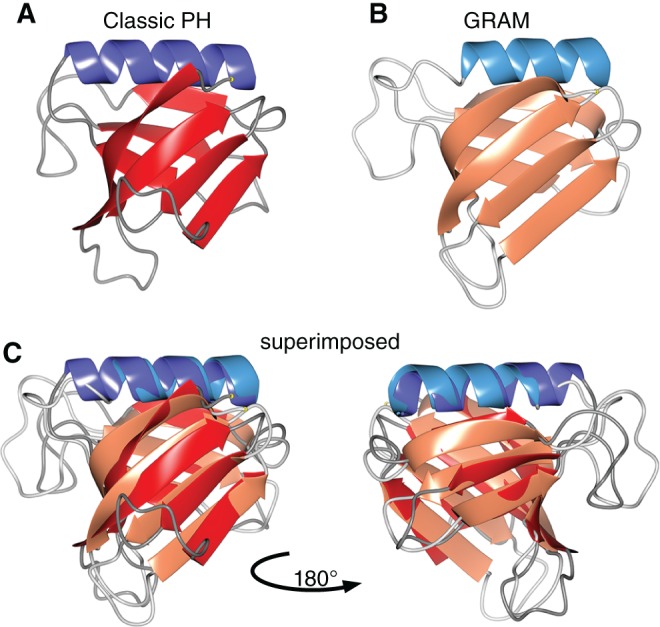
PH‐like domains that share no significant sequence have highly similar folds. Ribbon diagrams of the Cα backbones of: A, classical PH domain in PEPP1 (1upq_A, residues 54–152); B, GRAM domain of MTMR2 (1lw3_A, residues 83–183); C, the 2 structures superimposed (root mean square distance = 1.9 Å across 86 residues) with views of both sides of the beta sandwich. Models colored by secondary structure: red = sheet, blue = helix; strong colors = classical PH, weak colors = GRAM. For more detail in A‐C, see Movies 1–3, respectively. Domains in pleckstrin were initially identified as a family of homologues ~100‐120 aa,^29^ and several structures were solved soon after.^15^ GRAM domains were identified as a family of sequences ~70 aa long,^30^ with the first solved structure identified as beta sheets 1–5 of a complete PH‐like domain.^18^

PH‐like domains are defined by a combination of structure and sequence alignment (Box 1). Different PH‐like domain families are not linked by sequence alignment. For example, classical PH domains and GRAM domains have very similar alpha‐carbon backbones (Figure 1 and Movies S1‐S3, Supporting Information), but no PSI‐BLAST search seeded with a classical PH domain makes a significant alignment (ie, there is no hit) with GRAM domains and vice versa. Each PH‐like family is presumed to have diverged from a single ancestral protein. In addition there is some evidence that the whole clan diverged from a prokaryotic origin.^26^ PH‐like families (Table S1) have been defined by a variety of profile‐sequence tools (Box 2), but given that there is a lag between discoveries and upgrading databases, we repeated these definitions.

Box 1 **Defining a PH‐like domain**

PH‐like domains are the fourth most common fold in the human proteome.^14^ Their 8 structural elements (βββββββα) span 100‐120 aa, making an orthogonal, slightly splayed beta sandwich (4 + 3) capped by the helix (Figure 1, Movies S1 and S2). Excluding redundant sequences, >200 PH‐like domain structures have been solved. Structures have confirmed earlier identifications made solely on the basis of remote sequence homology in most^31^, ^32^ but not all^33^ cases.

The definition of a PH‐like domain we use is **either** (1): by structural similarity to the region occurring twice in pleckstrin. The peptide backbone follows the characteristic βββββββα‐pattern closely; **or** (2) by significant sequence homology to any domain that fits the structural definition. This may require indirect alignment (profile‐sequence) rather than just sequence‐sequence. Both the classical PH domain in PEPP1 and the GRAM domain in MTMR2 meet definition 1 (Figure 1, Movies S1‐S3), while in yeast the classical PH domain in Boi1p and the GRAM domain in Ymr1p (orthologous to MTMR2) meet definition 2.

Finding that a sequence has homology to a PDB entry documented as having a PH‐like fold goes some way to meet definition 2. However, PDB entries may contain multiple different domains, so it is important to check that a sequence shares homology with the PH‐like domain portion of the PDB sequence.

Box 2 **Profile‐sequence tools currently used to define domains**

All profile‐sequence tools are seeded with one or multiple sequences to find new *hits*, these being sequences that align more closely than the *E‐value* threshold. The *profile* is a statistical representation of the different residues across the multiple sequence alignment (MSA) made from seed plus hits. The profile is then used to initiate another round of searching, and because it contains more sequence information than the previous round it may detect more hits. The whole process iterates through multiple rounds until no more sequences are added.

Profile‐sequence tools differ in choice of seeds, in particular how broad a net to cast. For example, the tool *SuperFamily* only uses seeds with solved structure, while *Pfam* starts with any recognizable domain.^34^, ^35^ Tools also differ in the curation of the profiles obtained, with Pfam using human intervention to group structurally related families into clans.^36^ Tools also vary in the way profiles calculate the MSA. The simple way, which minimizes computation, scores all alignments using fixed gap opening/extension penalties. A more sensitive, but computationally harder, technique is to convert the MSA into a hidden Markov model (*HMM*), which codes insertions or deletions at each position in a profile‐specific way.^9^, ^37^ HMM profile‐sequence searches are used in Pfam and Gene3D.^38^, ^39^

Domain definitions are updated regularly (eg, Pfam now version 30, where the PH domain clan is CL0266, see pfam.xfam.org/clan/CL0266), but new discoveries are not disseminated to databases instantly.^40^ Upgrades lag behind the literature and curation is not perfect. For example, Pfam describes a domain in unconventional myosins as “Myosin_TH1,” but lacks any link to the PH‐like fold.^32^, ^41^, ^42^ In addition, some domains that do not meet the PH‐like definition because homology is too weak are nevertheless accepted. The basis for this is typically that the remainder of the protein sequence is highly homologous to a protein that does contain a PH‐like domain, and it is thought that the 2 proteins are full‐length homologues. For example Syt1p has a Sec7‐homology guanine exchange factor (GEF) domain, and related GEFs in other species have a classical PH domain. The non‐significant hit in Syt1p (E‐values: SMART = 0.002, Pfam not found (>10), SuperFamily = 0.05) has been accepted as PH‐like, because the GEF domain homology strongly enhances the likelihood of a PH‐like domain existing.^43^, ^44^

Yeast is one of the most highly annotated organisms. The Saccharomyces Genome Database (SGD) is annotated by InterProScan, which is a resource from the European Bioinformatics Institute (Hinxton, UK). InterProScan incorporates all of Pfam, SuperFamily, Gene3D and many other profile‐sequence tools.^45^, ^46^ For PH‐like domains in yeast, InterPro provides useful data, but its output is not close to 100% sensitive, only including 61 of 73 domains (Table S2).^47^, ^48^ Also, it is not entirely specific, as it includes 3 false positives (Table S2).

3

3.1

**Figure 2:**
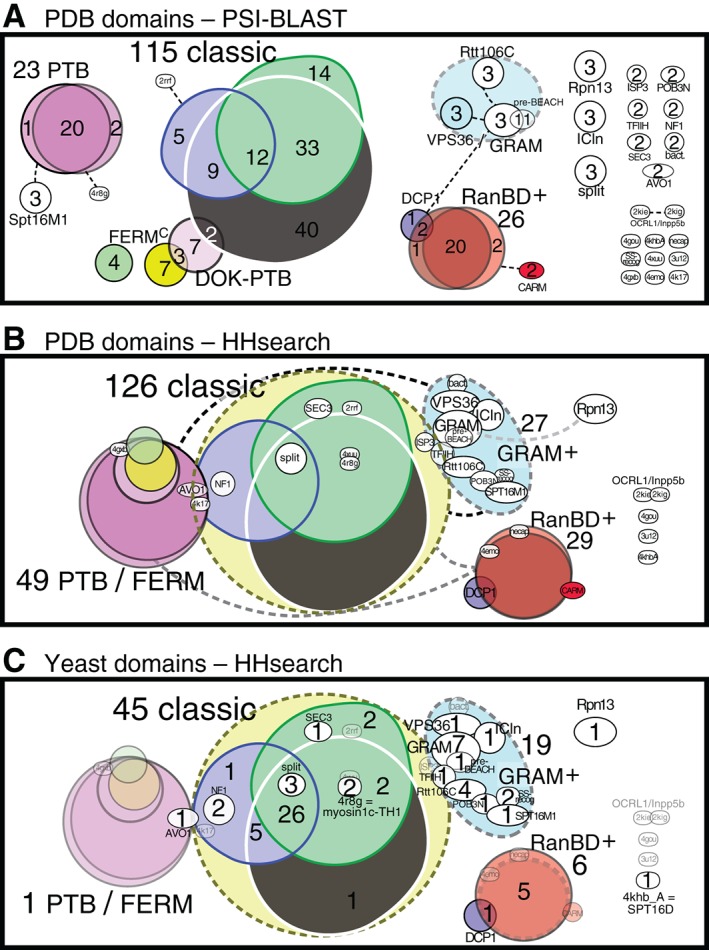
Family grouping of PH domains in PDB and yeast. A, ~240 PH‐like domains in PDB were divided into 39 families by PSI‐BLAST. The size of the colored shapes and extent of overlap correlates with numbers of domains. Family names are standard abbreviations, and PDB identifiers are listed in Table S1. Weak homologies, shown by dashed lines, were deduced from the presence of non‐significant hits (*P* > .005) that occurred with no false positive hits above them in hit lists. They lead to weak family grouping indicated by dashed outlines. Classical PH domains (~50% of all domains) are made up to 3 overlapping groups, and they have some overlap with some phosphotyrosine binding (PTB) domains. A high degree of overlap is seen within the 2 PTB groups and in the 2 Ran‐binding domain (RanBD) groups (EVH1, WH1). Twenty independent families contain 1, 2 or 3 domains with no links to larger families. B, The same domains and families were analyzed by HHsearch. Domains that produce hits to each other with prob[SS] ≥ 85% are grouped together. The 16 of 20 unlinked small families are now included in larger groups. PTB are fused with FERM‐C domains; also GRAM domains are fused with 9 other families. Some domains produce hits to both classical PH domains and either PTB/FERM‐C domains or GRAM domains, leading to partial fusion of these 3 groups. Dashed lines indicate incomplete overlap (prob[SH] = 50‐80% varying color grey to black). C, The families arranged according to the HHsearch analysis from (B) were populated with 73 yeast PH‐like domains described in the literature (details in Table S2A). Empty families are shown in faint outline.

Defining PH‐like domains starts with mining the literature, the PDB for solved structures, and sequence databases. For a standard definition of the PH‐like clan we used PSI‐BLAST to study PDB, which contains >200 different PH‐like domains. We described the minimal set of 39 PH‐like sequences that identified the complete set of PH‐like domains in PDB as strong hits in PSI‐BLAST searches. This minimal set indicates the presence of 39 families in the PH‐like clan (Figure 2A and Table S1). This classification is more complete than definitions of the PH‐like clan in databases (Box 2). The largest family consists of the classical PH domains (~50% of all sequences). Three families have ~10% of the domains each: FERM‐C, PTB and RanBD. Non‐significant hits indicated that some families share weak homology (Figure 2A). However, the main feature was the presence of 20 small families (≤3 members) that were completely isolated from all others (Figure 2A). The lack of cross‐detection between most families indicates that if other PH‐like domains existed, they might well be undetectable by PSI‐BLAST.

### 2.2 HHsearch: more sensitive than PSI‐BLAST

Profile‐profile tools such as HHsearch (Box 3) have previously detected many new domains,^49^, ^50^, ^51^, ^52^, ^53^, ^54^, ^55^, ^56^, ^57^, ^58^, ^59^ so we asked if HHsearch can improve on PSI‐BLAST for PH‐like domains. We first studied how HHsearch detects PH‐like domains in PDB, where we are certain if hits are true or false. This allowed us to determine key parameters relating to both specificity and sensitivity. Importantly, for this exercise HHsearch was set to be unaware of solved structure. HHsearch was seeded with sequences from our minimal set of 39 PH‐like families.

Box 3 **Profile‐profile searches with HHsearch (*HHpred*).**

HHsearch is freely accessible profile‐profile search software that is run either offline, or on a webserver called HHpred. The program is powerful,^13^ fast and flexible, with many settings that can be varied by users.

HHsearch has 3 stages^9^ (1): starting with a single seed or aligned multiple seeds (“query”), MSAs are made by multiple iterations of *HHblits*, which is more sensitive than PSI‐BLAST.^60^ (2) PSIPRED predicts 1 of 3 structural states (helix = H, sheet = E, loop = C) for each position in the MSA.^12^ (3) the query HMM containing both sequence and predicted secondary structure is used to search through pre‐made libraries of target HMMs built before‐hand. Freely available libraries (at http://toolkit.tuebingen.mpg.de/) include both “knowns” such as PDB, and curated domains from Pfam, SuperFamily, Gene3D(CATH) and other profile tools, and also “unknowns,” ie, proteomes, both prokaryotes (>30) and eukaryotes (x9): human, mouse, fruit fly, nematode, malarial parasite, thale cress and 3 fungi: budding yeast (*S. cerevisiae*), fission yeast (*S. pombe*) and maize blast (*U. maydis*). The 2 libraries used mostly for this study are PDB, which contains ~35 000 entries (non‐redundant at 70% sequence identity) updated weekly, and yeast, which has 5870 entries. A third library constructed in‐house was used for off‐line searches (see *Yeast‐to‐PDB* section and *Materials and Methods*).

Important variables that can be set by the user for each of the 3 stages are:

- **number of iterations.** The defaults number of iterations for building the query MSA is 3. Libraries of targets have been built with 2 iterations. Iterating more times (maximum number = 8) enhances sensitivity, but takes longer. Searches designed specifically to identify only orthologues within a large family of homologues may benefit from using 1 iteration, deliberately reducing sensitivity.
- **scoring secondary structure.** There is an option to not score secondary structure at all. Run off‐line, it is also possible to vary the proportion of the overall alignment weighted to secondary structure. Default is 11%, ie, sequence alignment dominates.
- **using solved structure.** If the PDB is being used as either query or target, the user can choose to use solved secondary structure states (reduced to 3 states) instead of predicted structure.

HHsearch returns up to ~20 000 hits ordered in terms of descending probability of shared structure (prob[SS]) with the query sequence. For each hit, apart from the prob[SS] value, there is a precise alignment (see Figure 5B) that can be summarized by 3 important values: (i) the statistical expectation (E‐value) of achieving the hit in terms of sequence alone given the size of the library, equivalent to the HHblits E‐value; (ii) the 2° structural similarity (2Ssim), which is independent of sequence homology; and (iii) the number of columns aligned between query and target (COLs), which can be as few as 6. We applied a length threshold that the alignment should cover 20% of the domain, ie, 20 aligned columns, and we found that shorter hits were often to a single structural element (Figure S1A). Hits that can be detected by PSI‐BLAST typically have a prob[SS] ≥ 99% and an E‐value of 10^−10^, which reflects the greater sensitivity of HHblits than PSI‐BLAST.

4

4.1

**Figure 3:**
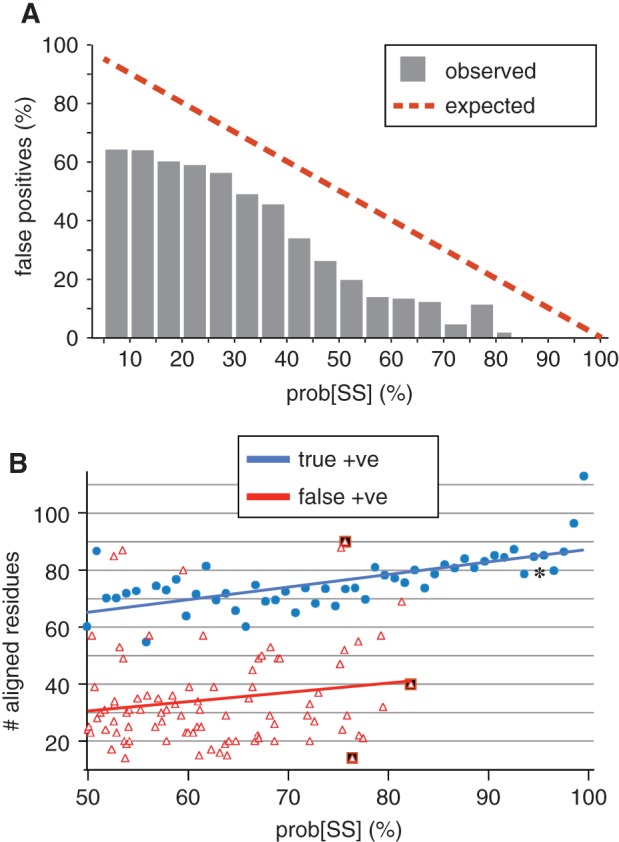
Properties of PH‐like and non‐PH‐like hits. A, Specificity of HHsearch at different levels of prob[SS]. Hit lists with prob[SS] ≥ 5% from 39 PDB‐to‐PDB searches were merged and scanned for the occurrence of non‐PH‐like domains. At each level of prob[SS], the rate of non‐PH‐like hits was less than predicted from the prob[SS] metric. No non‐PH‐like domain scored prob[SS] > 85%. B, The relationship between prob[SS] (≥50%) and the number of aligned residues (COLs, see Box 3), both for true positives (showing means for every prob[SS] centile (total n = 2200, median 22 hits per centile) and for non‐PH‐like hits (showing 90 individual occurrences). In both groups COLs increased with prob[SS]. Although COLs was lower for false positives, there were some exceptions. Non‐PH‐like hits similarly tended to have lower secondary structural similarity scores (data not shown). Three strong false positives (black squares) are described in detail in Figure S1. Asterisk indicates the position of Age1p, a strong false positive in yeast (Figure S1).

**Specificity (avoiding false positives):** HHsearch provides a list of alignments with decreasing probability of shared structure (prob[SS], Box 3). Previous work with HHsearch has found that a threshold of prob[SS] ≥80% is sufficient to detect many true positives.^49^, ^50^, ^51^, ^52^, ^53^, ^54^, ^55^, ^56^, ^57^, ^58^, ^59^ In our 39 searches, 1300 hits scored prob[SS] ≥80%, of which 1298 were PH‐like domains. Among all non‐PH‐like hits scoring prob[SS] ≥5%, the observed error rate was far less than indicated by prob[SS] (Figure 3A). This indicates that the prob[SS] metric is a conservative estimate of accuracy. Alignments to non‐PH‐like domains differed from true positives by being shorter (Figure 3B). In addition, non‐PH‐like domains had weaker secondary structural alignment (data not shown). These alignments were either to a single PH‐like structural element, or to longer portions of the PH‐like structure (Figure S1). We conclude from this benchmarking that a threshold prob[SS] ≥85% will ensure close to 100% specificity (Figure 3A).

**Sensitivity (avoiding false negatives):** To compare the sensitivities of HHsearch and PSI‐BLAST, we compared their ability to join the PH‐like clan together. The 39 searches with HHsearch produced 1200 hits to PH‐like domains in PDB with prob[SS] ≥ 85%, which is many more positive hits than PSI‐BLAST produced (n = 350). While 20 families were unlinked by PSI‐BLAST, HHsearch left just 4 unlinked families (Figure 2B). For example, the myosin 1c TH1 domain, isolated from other famiiies by PSI‐BLAST, produced a hit to classical PH domains with prob[SS] = 96%. HHsearch even linked GRAM domains to classical PH domains (Figure 2B). The detection by HHsearch of homologies that PSI‐BLAST could not find indicates that it might also to detect new PH‐like domains in proteins of unknown structure.

## 2.3 PDB‐to‐yeast: easy detection of some new domains

We next turned from searching PDB, where structure has been solved, to searching the yeast proteome where we have no structural information for 10%‐13% of 5900 proteins. Thorough curation of current literature identified 73 PH‐like domains in yeast. We noted that current database annotations lag behind this, containing 48 – 62 (66%‐85%) of these domains (Table S2A).

We used HHsearch to search a target HMM library containing all yeast proteins with the 39 PH‐like sequences defined above. Among hits with prob[SS] ≥85%, there were 71 of the 73 known yeast PH‐like domains (97%, Figure 2C). In addition, there were 5 hits with prob[SS] ≥ 85% to regions not previously identified as PH‐like. To investigate these predictions we turned to sequence homology. This might appear paradoxical, since by definition these regions have not been detected by profile‐sequence tools. However, these profiles were typically seeded on mammalian sequences, which biases the profile (Box 4). Thus, more information may be obtained about yeast proteins if they are used to seed profiles. We seeded PSI‐BLAST with all 5 regions. Four sequences from Caf120p, Lam1p, Sip3p and Vid27p produced top hits that were PH‐like domains, and other tools also identified these regions as PH‐like (Table S2B). Therefore, our PDB‐to‐yeast searches identified 4 new PH‐like domains.

Box 4 **Asymmetry in searches (Q: “How do I get where I want to go?” A:“I wouldn't start from here.”)**

Profile‐profile tools in a trivial way might be symmetric. So long as profiles are made the same way for use as queries and targets, comparing them either way round (query → target or target → query) makes no difference. On the other hand profile‐sequence tools are dependent on where they start because the profile represents a large group of sequences centred on the query. Thus, a profile‐sequence tool searching in 1 direction (eg, known‐to‐unknown) will probably not make the same hits as the same tool searching in the other direction (eg, unknown‐to‐known).

One way to overcome the problem of profile bias in favor of where it starts is to manipulate the growing MSA to attempt to eradicate any trace of its origin. HHblits does this by filtering an MSA (thousands of sequences) to only pick a small group (~100) that are chosen to maximize diversity.^60^

**Figure 4:**
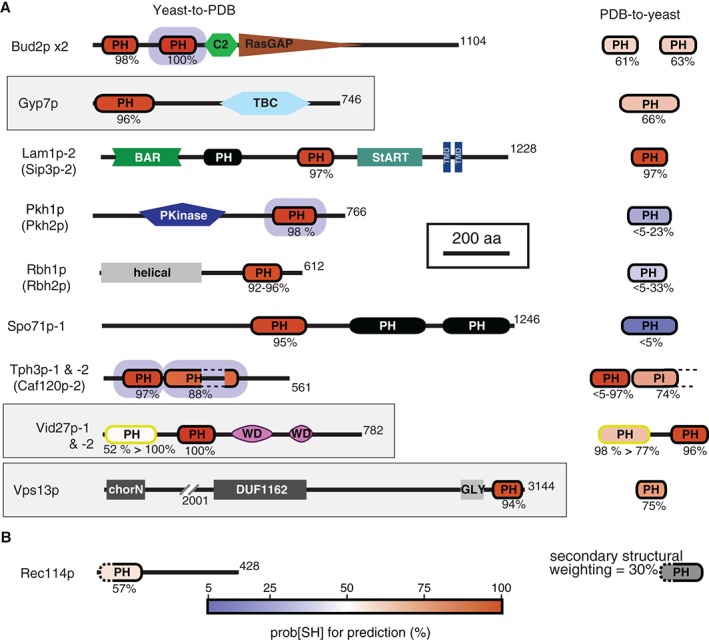
New PH‐like domains identified in yeast. A, 16 strongly predicted new PH‐like domains in yeast, shown in the context of 9 full‐length proteins. Four paralogs that share highly similar domain patterns have been omitted. New domains (black outline) are shaded according prob[SS] of strongest hits (blue–red graded scale). Main section shows yeast‐to‐PDB hits; right‐hand section shows PDB‐to‐yeast hits. Where paralogs are reported in the same line, both prob[SS] values are reported, but the diagram and shading belong to the hit with higher prob[SS]. For the 1 domain predicted by indirect alignment (yellow outline, Vid27p‐1), prob[SS] values are given for both searches. Light grey boxes (Gyp7p, Vid27p Vps13p) indicate new PH‐like families present widely in eukaryotic evolution. Bud2p‐2, Pkh1/2p, Tph3p and Caf120p (domains with green surrounds) have homologues with PH‐like domains at the same position, so these new discoveries are to some extent expected. Accompanying domains include other, known PH‐like domains (in Lam1p/Sip3p and Spo71p ‐ shaded black) as well as C2, RasGAP, DH, TBC, BAR, StART, and others as follows: domain of unknown function = DUF; transmembrane domain = TMD, protein kinase = PKinase, chorein‐N domain in VPS13 = chorN, WD40 = WD, glycine‐rich = GLY. The N‐terminus of Rbh1p (and Rbh2p) contains a helical domain of unknown function with homology to RhoGEFs. B, One PH‐like domain tentatively identified with low prob[SH]. This was first found with PDB‐to‐yeast searches using increased secondary structural weighting, hence the usual prob[SS] value scale does not apply. Details of all newly predicted PH‐like domains are in Table S2B.

The fifth region identified was at the N‐terminus of the ARF‐GEF Age1p. When this was seeded into HHsearch or other tools there was no match to PH domains or any other strong hit (Table S2B). Therefore, it is a false positive hit. We next investigated how such a strong false positive arose (asterisk in Figure 3B). We found an explanation in the way the MSA for Age1p was created. The MSA was made for full length Age1, which aligns with >100 other ARF‐GEFs. The N‐terminus of the MSA is spuriously dominated by PH‐like domains because of 3 factors: (1) the N‐terminus of Age1p is essentially unique; possibly it contains an defunct relic of a PH domain. (2) most of the other ARF‐GEFs in the MSA have a classical PH domain upstream of their GEF domain. (3) the Age1 N‐terminal region shares non‐significant sequence features with parts of 2 of these PH domains. This led to the inclusion of 2 genuine PH sequences in the MSA, even though they are divergent from Age1p (Figure S2). The N‐terminus of the Age1p MSA therefore aligns with classical PH domains. This kind of false positive can be avoided by repeating all positive alignments obtained by PDB ⇔ whole protein with just the identified domain (ie, PDB ⇔ domain). To do this, we made MSAs for all 5 newly identified regions with minimal flanks (3‐5 aa), and carried out pairwise alignments with the relevant PH‐like domains using *HHalign* (see *Materials and Methods*). This confirmed that only the 4 new regions in Caf120p, Lam1p, Sip3p and Vid27p are PH‐like domains (Figure 4A).

### 2.4 Yeast‐to‐PDB: a step change in sensitivity

A key finding in this work is that initiating a tool such as PSI‐BLAST with yeast sequences strongly matched them to defined PH‐like domains, even though the same hits were not seen in the other direction with PSI‐BLAST or any other profile sequence tool. This indicates that profiles centred on yeast sequences may contain critical information that is missed by profiles seeded on non‐yeast sequences. Since HHsearch makes profiles of both query and target, its results are potentially symmetrical (Box 4). However, standard usage of HHsearch is not entirely symmetrical. Profiles for queries use more iterations (up to 8, see Box 3). By comparison, target libraries were constructed with 2 iterations, and the model organism libraries were made several years ago, when there were fewer solved sequences and fewer sequenced divergent genomes. Since PSI‐BLAST benefits so much from being initiated on yeast sequences, we asked if HHsearch would also benefit from being seeded with yeast sequences.

We remade the entire yeast MSA library (see *Materials and Methods*). We then used each of the yeast MSAs in turn to search the PDB library. This identified 88 yeast regions where the top hit was **both** (1) a solved >200 PH‐like domain **and** (2) had a prob[SS] ≥ 85%. This group contained 72 of the 73 known yeast PH‐like domains (sensitivity = 99%). The 73rd known domain also matched PH‐like domains in PDB, but at prob[SS] = 79%. The 16 other regions included all 4 of the new regions found above and 12 further candidate PH‐like domains, with matches in the range of prob[SS] 88%‐100%. We tested if these were true positives by carrying out pairwise alignments. These showed strong hits to known PH‐like domains for 11 of the regions. One other region, in Pib2p, was a false positive for the same reasons as Age1p, in that matches to PH‐like domains were only obtained with longer segments (eg, the whole protein) not the domain alone (data not shown). We predict that the remaining 11 matches are all PH‐like domains (Figure 4A). Three of the proteins have no previous structural information: Yjl016wp, is a twin PH protein, the 3 ^rd^ in a family also containing Caf120p and Skg3p, so we named it Tph3p, for twin PH domain‐3. Yjl181cp and Yjr030cp are paralogs that are distant Ran binding domain homologs, so we named them Rbh1/2p.

Overall, seeding HHsearch with yeast proteins led to a clear increase in sensitivity for HHsearch. In practice, to obtain part of this benefit users could produce a list of unknowns enriched for true positives by using the hits with prob[SH] < 85% in PDB‐to‐yeast searches (Figure 3A). Among the 11 domains we found by reverse yeast‐to‐PDB searches, 5 had moderately high prob[SS] (>60%) in the more easily achieved PDB‐to‐yeast searches. These would require little work to find by yeast‐to‐PDB searches among a very small number of false positives. However, 4 of the 11 domains had prob[SS] < 5% in PDB‐to‐yeast searches, meaning that they would be missed from an enriched list. This indicates that high levels of sensitivity might require the construction of entire libraries, as we have done.

## 2.5 Indirect use of HHsearch for maximum sensitivity

One way to enhance the sensitivity of HHsearch without having to construct a library is to define intermediate sequences that are strong hits to both a PDB structure and to a yeast region of unknown function. Linking an unknown to the gold‐standard database indirectly is called transitive searching,^61^, ^62^ and theoretically it involves a massive increase in computation. For PH‐like domains in budding yeast, we carried out a simplified form of indirect searching by finding all PH‐like homologues for the 88 yeast PH‐like domains in 2 other fungi: *U. maydis* and *S. pombe*. Proteome‐wide libraries of these species are available within HHsearch. Using these fungal domains, we identified 1 extra PH‐like domain (Vid27p‐1, Figure 4A). Importantly, these indirect searches strongly confirm all 11 of the new domains that we found by yeast‐to‐PDB searches (data not shown).

Overall, HHsearch increased the number of PH‐like domains in yeast by >20% (73 → 89). Whether this involved offline use of HHsearch, or a combination of strategies for the online server HHpred (Workflows described in Box 5), maximum sensitivity required indirect searches. The newly described sequences add 5 further families to the PH‐like clan. Three of the new families are widely distributed throughout evolution, and all of these are involved in intracellular traffic: (i) Gyp7, late endosomal RabGAP in all eukaryotes including 5 human homologs: TBC1D15/16/17 RUTBC1/2; (ii) Vid27, vacuolar import and degradation in fungi and plants; (iii) Vps13, a membrane contact site protein affecting lipid metabolism in all eukaryotes (Figure 4A). Thus, predictions in yeast can have broad significance.

Box 5 **Workflows in HHpred**

**A. To find a fold/function for an unknown protein:**

1. Seed HHpred with the protein, choosing PDB and Pfam as targets. All hits above a fairly generous threshold (prob[SS] > 50%, COLs > 20) are worth considering further.
2. Seed searches with domain only to exclude false positives (Figure S2).
3. For every interesting region, confirm the significance of the hit by comparing its strength to that obtained by the strongest false positives in PDB‐to‐PDB searches (set to detect predicted structure only).

**B. To maximally extend the structural homologues for a known fold in a proteomes with an HMM library**

1. Use PSI‐BLAST to identify one representative sequence per family within the overall clan.
2. Use the representative sequences in PDB to PDB searches by HHsearch to set a threshold for prob[SS] that avoids false positives (with HHsearch option set to ignore known structure, and use predicted secondary structure).
3. Seed PDB‐to‐unknown HHsearch searches with these sequences, looking in the proteome of choice, identifying positive hits with the threshold obtained above.
4. Investigate non‐significant hits, especially those with relatively high COLs and 2Ssim (Box 3), by carrying out unknown‐to‐PDB searches.
5. Make more links between non-significant hits and PDB entries by indirect searches in related organisms. First, use unknown‐to‐related proteome searches to identify homologous regions in the related proteome. Use these hits to carry out related proteome‐to‐PDB searches.
6. Confirm all new matches with domain only searches.

A more complex alternative for the confirmatory steps (A2 and B6) is to create HMMs for the query and target regions with 8 iterations, and align them pairwise with HHalign.^63^

The final option we explored was to test different settings within HHsearch. To identify conserved folds with highly diverged sequence, we raised the secondary structure weighting (ssw) within each alignment. In HHsearch, this has a default of 11% (Box 3). Increasing ssw to 30% produced no major improvement in overall accuracy in identifying new PH‐like domains in yeast (data not shown). However, it led to 1 extra region in Rec114p being identified weakly, although it was below the false positive threshold. In yeast‐to‐PDB searches, the top hit for this region in PDB was a PH‐like domain, so this is a possible new PH‐like domain (Figure 4B).

### 2.6 Support for the predicted PH‐like domain in Vps13p

**Figure 5:**
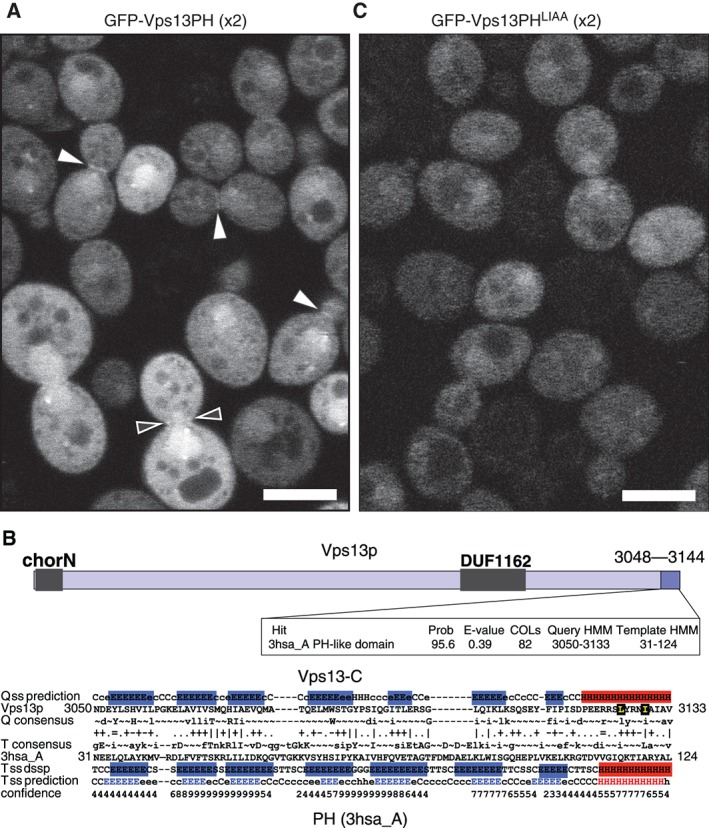
Intracellular targeting by the PH‐like domain of Vps13p. A, GFP‐Vps13‐PH‐PH (dimer) weakly targets the bud neck, seen as linear targeting across the neck of small‐to‐medium buds (filled arrowheads), and dots either side of occasional larger buds (hollow arrowheads). The minor nuclear enrichment is nonspecific, being seen with all other PH monomers and dimers (data not shown). B, Vps13p 3028–3144 as query (Q, top 3 lines) aligned with a target (T) hit from the solved structure 3hsa_A, a bacterial protein (*Shewanella amazonensis*, bottom 5 lines). The fourth line indicates which residues align, where: “|” is a very good alignment, “+” is good, “.” is neutral, “‐” is bad, and “=” is a clash. For both Q & T, the secondary structure prediction (ss prediction) is in 3 states, E for sheet (blue), H for helix (red) and C for unstructured loop (black), with prediction confidence shown for target. The target also has “ss_dssp” showing its solved structure. The box above shows statistics on the hit, including prob[SS] and COLs. L3125 and I3129 (highlighted in yellow) are partially conserved residues (lower case in consensus) that align with 3hsa_A ( “+” and “|,” respectively). Alignment made by HHalign. C, GFP‐tagged dimeric Vps13‐PH(LIAA) (L3125A and I3129A) accumulates in cells to a much lesser extent than wild‐type. Scale bars 5 µm.

One of the most typical functions of PH‐like domains is organelle targeting, binding proteins or lipids. In a previous survey of 33 yeast classical PH domains, only 9 had this function,^64^ but it is also applicable to other PH‐like families.^65^ To determine if the newly described PH‐like domains target membranes, we expressed GFP‐PH fusions for domains in 8 proteins implicated in membrane traffic/function: Bud2p (both domains), Gyp7p, Lam1p, Pkh2p, Tph3p (both domains), and Vps13p (Figure 4A). We also expressed the false positive in Age1p. None of these showed any specific cellular localisation (data not shown). We next constructed dimers (in the form GFP‐PH‐PH) for 6 of these PH‐like domains (all except Tph3p) and Age1p, since dimers can reveal weak membrane targeting.^66^, ^67^ All constructs remained diffusely cytosolic except for the Vps13 PH‐dimer, which faintly localized to rings at the bud neck of some cells (Figure 5A). This suggests that the region we identified in Vps13p is involved in intracellular targeting, and may be functionally important.

Full‐length Vps13p (3144 aa) has intracellular localisations to multiple membrane contact sites, including vacuole and mitochondrial patches (vCLAMP), endosome‐mitochondrial contacts and nucleus vacuole junctions (NVJ),^68^, ^69^, ^70^ but as yet no specific targeting domains have been tested. We determined the role of the predicted PH‐like domain by constructing a mutant version predicted to inactivate it. A desirable strategy would be to mutate putative ligand binding sites in the predicted variable loops sited at β1‐β2, β3‐β4 and β6‐β7.^64^ However, Vps13p has no conserved residues in these loops (Figure 5B). Therefore, we constructed 2 other mutants: (a) Vps13ΔPH, lacking the entire PH domain (deletion of 3029‐3144); (b) a double point mutation of conserved hydrophobic residues in the predicted alpha helix (“LIAA” = L3125A I3129A, Figure 5B). We predicted that these hydrophobic residues would stabilize the PH domain core, similar to the WxxxØ motif (Ø = hydrophobic residue) in classical PH domains, so the LIAA mutant may partially unfold.^71^ Cells expressed the PH(LIAA) dimer construct at a lower level than wild‐type dimer (Figure 5C). This is consistent with reduced protein stability caused by partial unfolding.

**Figure 6:**
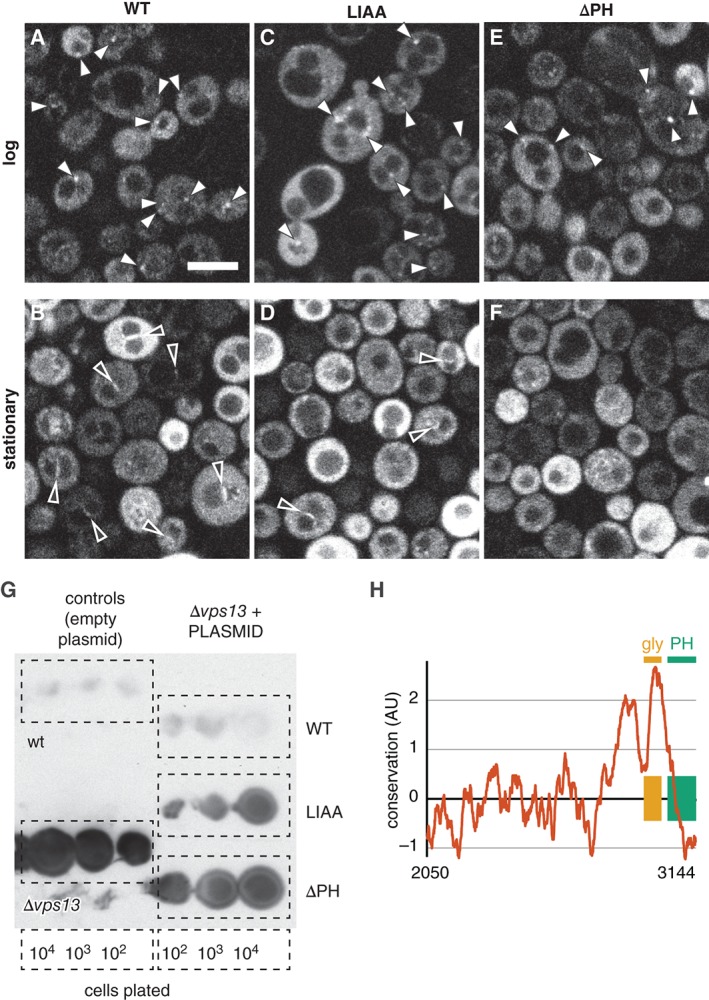
Intracellular targeting by PH‐like domain of Vps13p. A‐F, Vps13‐EGFP constructs, A/B: wildtype (WT), C/D: L3125A I3129A (LIAA) and E/F: 1–3028 (ΔPH) in *Δvps13* cells in log phase (A/C/E) or early stationary phase (B/D/F). Sites of localization include intracellular puncta (filled arrowheads) in log phase, and the nucleus vacuole junction (NVJ, open arrowheads) in stationary phase. Scale bar (shown in A only) 5 µm. G, Overlay blots to detect secreted CPY. Controls: wild‐type cells and *Δvps13* cells show no secretion and maximal secretion, respectively. Rescue of *Δvps13* was tested for Vps13‐EGFP plasmids as in A‐F. Results are representative of 6 similar experiments. H, Residue conservation in the C‐terminal 1095 residues of Vps13p, calculated by ConSurf and scaled as described in *Materials and Methods*. The most conserved part of the Vps13 C‐terminus is a glycine rich domain (orange) of no known function,^72^ not the predicted PH domain (green).

We next studied the effect of the two Vps13 mutations on intracellular distribution. Vps13‐EGFP showed a complex intracellular distribution: in log phase it was largely diffuse, with the majority of cells containing puncta, some of which were close to the vacuole (Figure 6A); by comparison in early stationary phase Vps13‐EGFP targeted the NVJ (Figure 6B). The two Vps13p mutants showed a marginal reduction in punctate targeting (Figure 6C/E), but considerable loss of NVJ targeting, which was partial for Vps13LIAA‐EGFP (Figure 6D) and undetectable for Vps13ΔPH‐EGFP (Figure 6F). Thus, the extreme C‐terminus of Vps13p appears to play a role in targeting, particularly to the NVJ in early stationary phase.

We next looked for a functional role of the proposed PH‐like domain in Vps13p. We tested if plasmid‐borne Vps13‐EGFP, Vps13LIAA‐EGFP and Vps13ΔPH‐EGFP rescue sorting of carboxypeptidase‐Y (CPY), a vacuolar enzyme that is subject to a strong vacuolar protein sorting (*vps*) defect (and hence is secreted) in *Δvps13* cells.^73^ Wild‐type Vps13 tagged at the C‐terminus with EGFP fully corrected the defect as shown previously.^70^ In contrast, Vps13ΔPH‐EGFP and Vps13LIAA in *Δvps13* produced significantly stronger signals than wild‐type Vps13‐EGFP, stronger for Vps13ΔPH‐EGFP than Vps13LIAA, although both were significantly weaker than empty plasmid (Figure 6G). Thus, both constructs in which the predicted PH‐like domain was mutated elicited partial rescue. This shows that the C‐terminus of Vps13p is functionally important, and this function requires the predicted PH‐like domain to be intact. Related PH‐like domains are widely predicted at the extreme C‐terminus of VPS13, for example in all 4 human homologs and in some plant homologs (data not shown), but this is not the most highly conserved part of the C‐terminus.^70^ Without the prediction by HHsearch, the PH‐like domain might be overlooked for an adjacent glycine‐rich domain that is far more conserved (Figure 6H).^72^

### 2.7 Beyond PH domains to the whole proteome

Is the increase in domain discovery seen for PH‐like domains likely to apply to other domains? To begin to examine this we surveyed 132 contiguous open reading frames on the right arm of yeast chromosome IV, representing 2% of the yeast proteome. MSAs for all ORFs were used to search PDB, and regions not previously known to contain domains were examined. Twenty‐one new domains were predicted in 16 (12%) of the proteins (Figure S3). As with PH‐like domains, many of these new domains would also be discoverable by systematically initiating PSI‐BLAST with the yeast sequence (prob[SS] ≥ 99% n = 9). Although some of the newly discovered domains do not strongly determine function (eg, the ARM repeat in Rrp1p), other predictions are more functionally significant (eg, intasome in Fob1p, vacuolar polyphosphate polymerase in Ydr089wp, a DNA‐binding domain in Ydr124wp). Seven newly predicted domains were found in the 19 proteins that had no prior structural/functional information, a discovery rate of 37%. Rolled out across the entire yeast proteome HHsearch could identify ~900 new domains, and 250–300 of these would be in the proteins that currently have no structural/functional information.

### 3 CONCLUSION

Current protein domain annotations can be significantly improved with profile‐profile tools, to catch up with advances in the literature, to focus searches on proteins of unknown function, and to maximize sensitivity. For the PH‐like clan, HHsearch identified the known domains and also extended their number by 20%. Elsewhere in yeast, application of HHsearch may be particularly good for proteins of unknown function. Yeast proteins are very good subjects for this tool because many organisms exist that diverged from it at different times in the last 10^9^ years, so homologues can be found at different evolutionary distances to provide information about functionally critical residues in yeast proteins. In the future we plan to analyze the yeast proteome systematically, annotating all the knowns and predicting many unknowns. Meanwhile HHpred is an extremely powerful online resource for discovering remote homologies.

### 4 MATERIALS AND METHODS

**Database files:** Files downloaded (source, date generated): InterProScan (SGD, December 2015), HHsuite 2.0.15 (Tuebingen Toolkit, June 2012) including yeast template HMMs (May 2008), PDB (“nr70,” ie, reduced in number so that no sequence shares more than 70% identity with any other, Tuebingen Toolkit), and Interpro 34.0.

**PSI‐BLAST:** Searches were carried out in 2 stages at Tuebingen Toolkit. Firstly the PDB sequence was seeded in searches of sequences from NCBI (nr70, *P* ≤ .005, iterations = 20). If new sequences were still added in the final iteration, a further round of 10 was initiated, until no more sequences were added (max 40 iterations, ≤4000 sequences). Aligned sequences were then submitted to a single round of BLAST using PDB as the target database.

**HHblits:** This was run on its own or as part of HHsearch with up to 8 iterations and an e‐value threshold for inclusion in MSA = 0.001–0.01.

**HHsearch:** Use was both online (“HHpred”) at http://toolkit.tuebingen.mpg.de 74 and locally in R. Settings were default except: 8 iterations of HHblits, “maximum accuracy” alignment was turned on, E‐value = 0.01, ≥1000 matches were returned without a lower limit on prob[SS].

To allow secondary structure to be weighted higher than the standard 11%, we edited line 853 of “hhhitlist.C”:

hit.score_aass = (hit.logPval < −10.0? hit.logPval: log(−log(1‐hit.Pval)))/0.45–fmin(lamda*hit.score_ss, fmax(0.0,0.2*(hit.score‐8.0)))/0.45–3.0

The underlined term was reduced to:

hit.score_aass = (hit.logPval < −10.0? hit.logPval: log(−log(1‐hit.Pval)))/0.45–lamda*hit.score_ss/0.45–3.0

This change removed a cap on the contribution of secondary structure.

**HHalign:** Domain sequences from both PDB and yeast proteins with 3‐5 flanking residues where available were submitted to HHblits, and the resulting “representative alignments,” deleting the secondary structure prediction and confidence entries, were compared in HHalign run with standard settings.

**Yeast HMM library (Ychunk200):** Total yeast protein sequence was obtained from downloads.yeast-genome.org/sequence/S288C_reference/orf_protein/. The problem highlighted by Age1p (Figure S2) was addressed by dividing proteins into regions of 200 residues (overlapping by 100), producing ~26 000 “chunks.” MSAs and HMMs were created for all chunks (8 iterations, ssw = 11%) to create a “Ychunk200” library that is available as an FTP download on request.

**Yeast‐to‐PDB searches:** All HMMs in Ychunk200 were submitted as queries for HHsearch using the PDB library (June 2013) as target. Top hits to PDB entries that contain PH‐like domains were then filtered for the presence of multiple domains, and if these were found alignments were curated by hand to include only those hits where the alignment includes the PH‐like domain.

**Residue conservation across the C‐terminus of Vps13p:** ConSurf was used with standard settings for the final 1095 aa of Vps13p.^75^ Results were normalized so that the 50th centile is 0 and the interquartile distance (75th to 25th) = 1. Values were smoothed by taking a rolling average of ×±9 (n = 19). To check that an internal domain did not force mis‐alignment at the extreme C‐terminus, the final 116 aa were submitted separately with no significant difference.

**Plasmids:** All plasmids were based on pRS416 (CEN, *URA3*), and used the 168 bp fragment from the promoter of *PHO5* for moderately strong, constitutive expression. PH domains were cloned as monomers in the form GFP—LGSAPVMAS—cloned sequence—SR*, and dimers were separated by either SR or SS. Residues used were Bud2p‐1 = 1‐163, Bud2p‐2 = 168‐319, Gyp7p = 1‐182, Pkh1p = 562‐674, Pkh2p = 833‐966, Tph3p‐1 = 1‐177, Tph3p‐2 = 180‐561, Vps13p = 3028‐3144, and Ysp1p‐2 = 568‐717. Full length and truncated Vps13p constructs were cloned by gap repair to clone either Vps13‐EGFP (1–3144 aa), Vps13ΔPH‐EGFP (1–3028 aa), or Vps13LIAA‐EGFP (1–3144, L3125A and I3129A). All PCR products and non‐gap repaired segments were checked by sequencing.

**Microscopy:** Cells grown at 30°C to mid log phase, or 16 h thereafter for early stationary phase, were immobilized between slide and coverslip, and visualized on an AOBS SP2 confocal microscope (Leica, Wetzlar, Germany) at room temperature, using identical settings for images comparing different constructs.

**Vacuolar Protein Sorting of CPY:** Haploid BY4741 yeast lacking *VPS13* were transformed with full length and mutated Vps13‐EGFP plasmids, as well as a plasmid expressing GFP‐GFP. The parent wild‐type strain, also carrying GFP‐GFP, was included as positive control. Ten‐fold dilutions of cells were spotted on selective medium (10^4^, 10^3^, 10^2^ per spot) and grown for 24 h before being overlaid with a nitrocellulose membrane for 16 h. This was washed extensively, and then processed to detect CPY,^73^ using monoclonal anti‐CPY (10A5B5, Thermofisher, Paisley, UK).

## CONFLICT OF INTEREST

The authors declare that they have no conflicts of interest.

## AUTHOR CONTRIBUTIONS

DRF adapted the program hhhitlist.C, constructed Ychunk200, and carried out all offline HHsearches. SEM assayed CPY sorting, and carried out cloning and imaging. KC created and analyzed match lists, and created display items. PA, RE‐T and JI created and analyzed match lists. TPL conceived the study, created match lists, and created display items. All authors contributed to drafting of the manuscript, and read and approved the final manuscript.

**Glossary**
**Clan:** a group of domain families that share the same fold but cannot be linked by standard sequence alignment tools (eg, PSI‐BLAST) alone. Sometimes called superfamilies.
**E‐value:** each alignment between query and target is assigned an e‐value, which is the number of hits as good as the one obtained that would be expected to occur randomly given the size of the database being searched. A threshold is chosen, (here 0.01) so that alignments more statistically unlikely than this are considered significant.
**HHalign:** pairwise application of HHsearch for direct comparison of 2 MSAs.
**HHblits:** Builds profiles from a single query via multiple rounds of searching, differing from PSI‐BLAST in several ways that increase speed and sensitivity.
**HHpred:** online server for HHsearch.
**HHsearch:** profile‐profile tool that explicitly weights a proportion of an alignment to aligned (predicted) secondary structure. MSAs can be built by either PSI‐BLAST or HHblits.
**Hit:** target in database that aligns with an e‐value more statistically significant (ie, lower) than the chosen threshold.
**HMM:** Hidden Markov Model: a way to represent MSAs, coding features including penalizing insertions and deletions in a profile‐specific way; more computationally complex than a simple profile.
**MSA:** multiple sequence alignment: sequences with multiple small regions of local homology are aligned across a large region by inserting gaps. MSAs can be dominated by large numbers of highly related sequences (eg, mammalian orthologues). This problem can be addressed by filtering MSAs for redundancy, reducing non‐diversity.
**Pfam:** a tool that identifies domains broadly, even without known structure/function. Pfam domains are constructed as HMMs. They are curated, reliable, and when structurally related families are found, they are grouped into clans on the basis of structural homology confirmed by HHsearch.^35^
**Profile:** statistical representation of the residues across an MSA, sometimes represented as a protein “logo.” Simple profiles apply inflexible, standard rules, for example for insertions/deletions.
**PSI‐BLAST:** Position‐specific iterated basic local alignment search tool. Builds profiles from a single query via multiple rounds of BLAST.
**Query:** (or seed) sequence from which searches are initiated.
**SuperFamily:** a narrow specificity tool for identifying domains similar to known structures, similar to SCOP.
**Target:** group of sequences curated into a database among which homologs are being sought. Database size varies from relatively small (all solved structures, predicted proteins in individual genomes) to very large (all proteins in all genomes). Size can be reduced by excluding redundant sequences sharing more than a pre‐determined level of sequence identity (20%‐90%).

## Supporting information

Table S1 Classifications of PH‐like domains. Information on our classification of 39 PH‐like families (see Figure 2), starting with the major groups that contain overlapping families in some classifications. Our classification is compared to those in Conserved Domain Database (CDD) at NCBI, the Structural Classification of Protein (SCOP) database at scop.berkeley.edu/, and Pfam 30.0. RED indicates PH‐like families that were missing from the PH‐like clan. “X” indicates that the family was missing completely. The penultimate column shows the number of PH‐like domains for each family in PDB (using a version non‐redundant at 70% identity), both for grouped families (excluding extensive overlap, n = 34) or the full list (n = 39). The final column shows the 1 structure we chose to represent the family for searches in PSI‐BLAST and HHsearch. Where the structure includes more than 1 PH‐like domain, the segment used is indicated. The bottom lines show the total number of PH‐like families in each classification, and information on 1 domain wrongly assigned as PH‐like in Pfam. Table S1 (with Figure 2A): compares previous and current categorizations of the PH‐like clan, and lists seeds used here.
Table S2. Description of PH‐like domains in yeast. A, 73 known PH‐like domains in yeast identified by 5 different methods: InterProScan (available through yeastgenome.org), Smart, SuperFamily (methods 1‐3) and HHsearch (PDB‐to‐yeast and yeast‐to‐PDB = methods 4 and 5). For methods 1‐3, any family specified is indicated (cPH = classical PH), while “+” indicates a generic PH‐like identification. § False positives in InterProScan were produced by Gene3D in Cbk1, Fpk1p and Kin82p. HHsearch identified Age1p as a false positive (see Figure S4). For method 4, the prob[SS] and family (among the 39 we defined: see Figure 2) of the strongest hit to the yeast domain is indicated. Other notes: c = crystal structure solved, h = homologous to a solved structure, o = omitted by Yu et al. (2004)^42^; ° numbering of PH‐like domains within individual proteins takes account of the new domains we identify (see Figure 4). B, Newly identified PH‐like domains in yeast. Details include discoverability by different tools: (1) PSI‐BLAST, showing E‐value (log_10_) and number of iterations (#) when a known PH‐domain (definitions 1 or 2 including yeast proteins in part A above) is found in the hit list; ^T^ indicates only temporarily in hit list, lost before convergence; (2) SMART domain prediction server, showing E‐values (log_10_) for any domains detected; (3) FFAS profile‐profile tool, (score is more significant when more negative, threshold = −9)^74^; (4) PHYRE2 structural prediction tool (showing probability of PH‐like fold, if top hit).^75^ HHsearch: method 5 = PDB‐to‐yeast (prob[SS]and query); method 6 = yeast‐to‐PDB (prob[SS] and target); method 7 = HHalign result with target in method 6. Prob[SS] values outlined in red were used to make identifications: 4 in PDB‐to‐yeast, 11 in yeast‐to‐PDB; 1 further identification was made by indirect searches (outlined in black). Other notes: ¶ Bud2p‐1 is indicated by SMART as overlapping a false‐positive C2 domain; § new true positive in Pkh1p already in SGD (identified by Gene3D); “T” indicates that for Pkh2p and Vid27p‐2 the PSI‐BLAST hit was only temporary, and was missing at convergence. Grey shading = non‐significant hit in Rec114p found using ssw = 30%; yellow = false positive for Age1p‐N, where the top hit in yeast‐to‐PDB searches was a thioredoxin, and pairwise alignment of Age1p‐N and the PH domain from centaurin (human ARFGEF) had prob[SH] = 0.1%. Table S2 (with Figures 2C and 4): A, compiles literature not previously available in 1 resource, and is background information for Figure 2C; B, shows detail for Figure 4.
Figure S1. Structures of strongest hits that are not PH‐like domains. Three of the strongest false hits to non‐PH‐like proteins among PDB‐to‐PDB HHsearches with 39 PH‐like domains (indicated as black squares in Figure 3A): A, C‐terminus of yeast Get5p (3vej_A) aligned at 14 of its 41 residues to the helix of 4chj_A, prob[SS] = 77%; B, mid‐section of human FANCL (3zqs_A) aligned at 40 of its 186 residues to strands 3‐5 of 2kig_A, prob[SS] = 83%. C, αβ subdomain of NS1 from Dengue virus type 2 (within a multi‐domain structure of 350 aa, not shown) aligned at 90 of its 114 residues to all 7 sheets in 4gou_A, prob[SS] = 76%. Aligned regions shown in colors (blue = helix, red = sheet, yellow = loop). While some non‐PH‐like hits are very short and can be identified as non‐PH‐like by that criterion (A), others cover multiple structural elements and cannot be excluded on the basis of shortness (B and C). Figure S1 (with Figures 1 and 3): shows worked examples from Figure 3B is a comparison with Figure 1.
Figure S2: Dissection of a false positive in Age1p. A, Alignment showing a strong hit obtained in HHsearch between the PH‐like domain of human ArfGEF Centaurin and the N‐terminus of Age1p. For interpretation of the alignment, see Figure 5B legend. Three unstructured loops in centaurin of 43, 7 and 8 aa are omitted. The single‐most conserved residue in PH‐like domains is the tryptophan in the helix (asterisk), which is reported as making a very good hit (|) with a glutamine in Age1p. B, All sequence from the first 102 columns of the Age1p MSA in HHsuite. This part of the MSA includes only 3 of 164 sequences: Age1p N‐terminus (1‐102/482aa), *Y. lipolytica* ArfGEF (GI:50550125; 735‐833/1099aa), and *T. nigroviridis* ArfGEF (GI:47212317; 425‐474/991aa). Coloring by HHsuite AlignmentViewer. All residues where the HHsearch alignment in part A. reported a good or very good hit (+ or |) are scored for actual conservation between Age1p and the other sequence(s): +, ±, − for strength of hit. Residues 81‐97 of Age1p predicted to align with alpha helix of centaurin in A. contains multiple prolines in its second half (highlighted in both A and B). The N‐terminus of Age1 has homologs only in species very closely related to *S. cerevisiae*, so this part of the MSA should only have 1 sequence. However, the downstream GEF (Age1p residues 168‐307) has 163 homologs, many of which have a domain structure BAR‐PH‐GEF. Two of these align to part of Age1p 1‐130 (A). These give a PH‐like appearance to the MSA at its N‐terminus that is not present in the original seed (B). Figure S2 (with Figures 3B and 4): shows detail on sole mechanism uncovered for false positives in HHsearch.
Figure S3. New domains found in 132 consecutive yeast genes. 132 consecutive verified open‐reading frames on the long arm of chromosome IV (“YDR”) starting at the centromere (YDR001C‐134C) were analyzed by HHsearch. Names in bold indicate proteins (x16) where new domains were documented (x21). New domains are outlined in black and shaded by prob[SS] on a white‐red scale; scores and main hit in PDB or Pfam are also given. All new hits were confirmed by pairwise alignments of domains alone. Asterisks indicate that the domain was detected by SMART even though absent from InterProScan data displayed at yeastgenome.org). Known domains in these proteins are shaded as in Figure 4 or in green. Naming: where a protein only has a systematic name, just the suffix is given (ie, “XYZ ^w/c^” from the full name YDRXYZw/c). Red indicates the proteins (19 = 14%) without prior structural/functional predictions (discounting assignment to a fungal protein family and prediction of transmembrane domains). Seven of these (37%) had newly predicted domains. By contrast, 9 of 113 (8%) previously characterized proteins had newly identified domains. Figure S3 (with Beyond PH domains section): shows the extent to which new domains can be found.

Movie S1. PH‐like domains share structure. Rotating models of structures in Figure 1. Accompany Figure 1.

Movie S2. PH‐like domains share structure. Rotating models of structures in Figure 1. Accompany Figure 1.

Movie S3. PH‐like domains share structure. Rotating models of structures in Figure 1. Accompany Figure 1.
