# Supplementary material for: Using HHsearch to tackle proteins of unknown function: A pilot study with PH domains
Source: Traffic. 2016 Oct 9;17(11):1214–26. doi: 10.1111/tra.12432 (PMC5091641; doi:10.1111/tra.12432)
Supplement: Supplementary file 1 — Table S1 Classifications of PH‐like domains. Information on our classification of 39 PH‐like families (see Figure 2), starting with the major groups that contain overlapping families in some classifications. Our classification is compared to those in Conserved Domain Database (CDD) at NCBI, the Structural Classification of Protein (SCOP) database at scop.berkeley.edu/, and Pfam 30.0. RED indicates PH‐like families that were missing from the PH‐like clan. “X” indicates that the family was missing completely. The penultimate column shows the number of PH‐like domains for each family in PDB (using a version non‐redundant at 70% identity), both for grouped families (excluding extensive overlap, n = 34) or the full list (n = 39). The final column shows the 1 structure we chose to represent the family for searches in PSI‐BLAST and HHsearch. Where the structure includes more than 1 PH‐like domain, the segment used is indicated. The bottom lines show the total number of PH‐like families in each classification, and information on 1 domain wrongly assigned as PH‐like in Pfam. Table S1 (with Figure 2A): compares previous and current categorizations of the PH‐like clan, and lists seeds used here. Table S2. Description of PH‐like domains in yeast. A, 73 known PH‐like domains in yeast identified by 5 different methods: InterProScan (available through yeastgenome.org), Smart, SuperFamily (methods 1‐3) and HHsearch (PDB‐to‐yeast and yeast‐to‐PDB = methods 4 and 5). For methods 1‐3, any family specified is indicated (cPH = classical PH), while “+” indicates a generic PH‐like identification. § False positives in InterProScan were produced by Gene3D in Cbk1, Fpk1p and Kin82p. HHsearch identified Age1p as a false positive (see Figure S4). For method 4, the prob[SS] and family (among the 39 we defined: see Figure 2) of the strongest hit to the yeast domain is indicated. Other notes: c = crystal structure solved, h = homologous to a solved structure, o = omitted by Yu et al. (2004)42; ° [file TRA-17-1214-s002.docx]

**Using HHsearch to tackle proteins of unknown function – a pilot study with PH domains**

David R. Fidler^1^*, Sarah E. Murphy^1^*, Katherine Courtis^1^, Pantelis Antonoudiou^1^, Rana El-Tohamy^1^, Jonathan Ient^1^ and Timothy P. Levine^1¶^

^1^Department of Cell Biology, UCL Institute of Ophthalmology, 11-43 Bath Street, London EC1V 9EL, UK

* these authors contributed equally to this work

^¶^ to whom correspondence should be addressed

Contact emails

Timothy P. Levine: tim.levine@ucl.ac.uk

**Supplemental Information**

**__**

**Supplemental Table 1. Classifications of PH-like domains.**

Information on our classification of 39 PH-like families (see Figure 2), starting with the major groups that contain overlapping families in some classifications. Our classification is compared to those in Conserved Domain Database (CDD) at NCBI, the Structural Classification of Protein (SCOP) database at scop.berkeley.edu/, and Pfam 30.0. RED indicates PH-like families that were missing from the PH-like clan. “X” indicates that the family was missing completely. The penultimate column shows the number of PH-like domains for each family in PDB (using a version non-redundant at 70% identity), both for grouped families (excluding extensive overlap, n=34) or the full list (n=39). The final column shows the one structure we chose to represent the family for searches in PSI-BLAST and HHsearch. Where the structure includes more than one PH-like domain, the segment used is indicated. The bottom lines show the total number of PH-like families in each classification, and information on one domain wrongly assigned as PH-like in Pfam.

**Supplemental Table 2. Description of PH-like domains in yeast**

**A.** 73 known PH-like domains in yeast identified by 5 different methods: InterProScan (available through yeastgenome.org), Smart, SuperFamily (methods 1–3) and HHsearch (PDB-to-yeast and yeast-to-PDB = methods 4 and 5). For methods 1-3, any family specified is indicated (cPH = classical PH), while “+” indicates a generic PH-like identification. § False positives in InterProScan were produced by Gene3D in Cbk1, Fpk1p and Kin82p. HHsearch identified Age1p as a false positive (see Supplemental Figure 4). For method 4, the prob[SS] and family (among the 39 we defined – see Figure 2) of the strongest hit to the yeast domain is indicated. Other notes: c = crystal structure solved, h = homologous to a solved structure, o = omitted by Yu *et. al*. (2004) ^(64)^; ° numbering of PH-like domains within individual proteins takes account of the new domains we identify (see Figure 4).

**B.** Newly identified PH-like domains in yeast. Details include discoverability by different tools: (1) PSI-BLAST, showing E-value (log_10_) and number of iterations (#) when a known PH-domain (definitions 1 or 2 including yeast proteins in part A above) is found in the hit list; ^T^ indicates only temporarily in hit list, lost before convergence; (2) SMART domain prediction server, showing E-values (log_10_) for any domains detected; (3) FFAS profile-profile tool, (score is more significant when more negative, threshold = –9) ^(47)^; (4) PHYRE2 structural prediction tool (showing probability of PH-like fold, if top hit) ^(48)^. HHsearch: method 5 = PDB-to-yeast (prob[SS]and query); method 6 = yeast-to-PDB (prob[SS] and target); method 7 = HHalign result with target in method 6. Prob[SS] values outlined in red were used to make identifications: 4 in PDB-to-yeast, 11 in yeast-to-PDB; one further identification was made by indirect searches (outlined in black). Other notes: ¶ Bud2p-1 is indicated by SMART as overlapping a false-positive C2 domain; § new true positive in Pkh1p already in SGD (identified by Gene3D); “T” indicates that for Pkh2p and Vid27p-2 the PSI-BLAST hit was only temporary, and was missing at convergence. Grey shading = non-significant hit in Rec114p found using ssw=30%; yellow = false positive for Age1p-N, where the top hit in yeast-to-PDB searches was a thioredoxin, and pairwise alignment of Age1p-N and the PH domain from centaurin (human ARFGEF) had prob[SH]=0.1%.

**Supplemental Figure 1: Structures of strongest hits that are not PH-like domains**

Three of the strongest false hits to non-PH-like proteins among PDB-to-PDB HHsearches with 39 PH-like domains (indicated as black squares in Figure 3A): **A.** C-terminus of yeast Get5p (3vej_A) aligned at 14 of its 41 residues to the helix of 4chj_A, prob[SS]=77%; **B.** mid-section of human FANCL (3zqs_A) aligned at 40 of its 186 residues to strands 3–5 of 2kig_A, prob[SS]=83%. **C.** αβ subdomain of NS1 from Dengue virus type 2 (within a multi-domain structure of 350 aa, not shown) aligned at 90 of its 114 residues to all 7 sheets in 4gou_A, prob[SS]=76%. Aligned regions shown in colors (blue = helix, red = sheet, yellow = loop). While some non-PH-like hits are very short and can be identified as non-PH-like by that criterion (A), others cover multiple structural elements and cannot be excluded on the basis of shortness (B & C).

**Supplemental Figure 2: Dissection of a false positive in Age1p**

**A.** Alignment showing a strong hit obtained in HHsearch between the PH-like domain of human ArfGEF Centaurin and the N-terminus of Age1p. For interpretation of the alignment, see Figure 5B legend. Three unstructured loops in centaurin of 43, 7 and 8 aa are omitted. The single-most conserved residue in PH-like domains is the tryptophan in the helix (asterisk), which is reported as making a very good hit (|) with a glutamine in Age1p. **B.** All sequence from the first 102 columns of the Age1p MSA in HHsuite. This part of the MSA includes only 3 of 164 sequences: Age1p N-terminus (1-102/482aa), *Y. lipolytica* ArfGEF (GI:50550125; 735-833/1099aa), and *T. nigroviridis* ArfGEF (GI:47212317; 425-474/991aa). Coloring by HHsuite AlignmentViewer. All residues where the HHsearch alignment in part A. reported a good or very good hit (+ or |) are scored for actual conservation between Age1p and the other sequence(s): +, ±, – for strength of hit. Residues 81-97 of Age1p predicted to align with alpha helix of centaurin in A. contains multiple prolines in its second half (highlighted in both A and B). The N-terminus of Age1 has homologs only in species very closely related to *S. cerevisiae*, so this part of the MSA should only have one sequence. However, the downstream GEF (Age1p residues 168-307) has 163 homologs, many of which have a domain structure BAR-PH-GEF. Two of these align to part of Age1p 1-130 (A). These give a PH-like appearance to the MSA at its N-terminus that is not present in the original seed (B).

**Supplemental Figure 3: New domains found in 132 consecutive yeast genes**

132 consecutive verified open-reading frames on the long arm of chromosome IV (“YDR”) starting at the centromere (YDR001C-134C) were analyzed by HHsearch. Names in bold indicate proteins (x16) where new domains were documented (x21). New domains are outlined in black and shaded by prob[SS] on a white–red scale; scores and main hit in PDB or Pfam are also given. All new hits were confirmed by pairwise alignments of domains alone. Asterisks indicate that the domain was detected by SMART even though absent from InterProScan data displayed at yeastgenome.org). Known domains in these proteins are shaded as in Figure 4 or in green. Naming: where a protein only has a systematic name, just the suffix is given (*i.e.* “XYZ w/c” from the full name YDRXYZw/c). Red indicates the proteins (19 = 14%) without prior structural/functional predictions (discounting assignment to a fungal protein family and prediction of transmembrane domains). Seven of these (37%) had newly predicted domains. By contrast, nine of 113 (8%) previously characterised proteins had newly identified domains.

**Movies 1-3: PH-like domains share structure**

Rotating models of structures in Figure 1.
